# Treatment with long-acting lanreotide autogel in early infancy in patients with severe neonatal hyperinsulinism

**DOI:** 10.1186/s13023-017-0653-x

**Published:** 2017-06-02

**Authors:** Heike Corda, Sebastian Kummer, Alena Welters, Norbert Teig, Dirk Klee, Ertan Mayatepek, Thomas Meissner

**Affiliations:** 1 Department of General Pediatrics, Neonatology and Pediatric Cardiology, University Children’s Hospital Duesseldorf, Moorenstrasse 5, 40225 Duesseldorf, Germany; 2 University Children’s Hospital, Katholisches Klinikum, Ruhr-Universität Bochum, Bochum, Germany; 3 Department of Diagnostic and Interventional Radiology, Medical Faculty, University Duesseldorf, Duesseldorf, Germany

**Keywords:** Congenital hyperinsulinism, Beckwith-Wiedemann syndrome, Hyperinsulinaemic hypoglycaemia, Somatostatin analogue, Adverse event

## Abstract

**Background:**

Treatment of severe diffuse congenital hyperinsulinism (CHI) without sufficient response to diazoxide is complicated by the lack of approved drugs. Therefore, patients are often hospitalized long-term or have to undergo pancreatic surgery if episodes of severe hypoglycaemia cannot be prevented. A long-acting somatostatin analogue, octreotide, has been reported to be an effective treatment option that prevents severe hypoglycaemia in children with CHI, and its off-label use is common in CHI. However, octreotide requires continuous i.v. or s.c. infusion or multiple daily injections. Here, we report our experiences with the use of a monthly application of a long-acting somatostatin analogue, lanreotide autogel® (LAN-ATG), in early infancy.

**Results:**

The mean blood glucose concentration within 7 days before the first LAN-ATG administration were compared to 7 days after the first LAN-ATG administration and increased by 0.75 mmol/L (range 0.39–1.19 mmol/L). In the following weeks intravenous glucose infusions, octreotide, and glucagon treatment could be successfully stopped in all patients 3–20 days after the first LAN-ATG injection without substantial worsening of the hypoglycaemia rate. Increased carbohydrate requirements could be normalized with an average reduction in the carbohydrate-intake of 7 g/kg body weight/d (range 1.75–12.8 g/kg body weight/d). Over a total of 52 treatment months, no serious adverse effects occurred.

**Conclusion:**

Long-term LAN-ATG treatment improved blood glucose concentrations, lowered the frequency of hypoglycaemia or allowed for normalization of oral carbohydrate intake in infants with CHI younger than 6 months of age. No severe side effects were observed. LAN-ATG might be an alternative treatment option in infants with severe CHI who lack risk factors for necrotizing enterocolitis and are not responding to current treatment regimens as an alternative to surgery after careful individual evaluation.

## Background

Congenital hyperinsulinism (CHI) is the most frequent cause of persistent hypoglycaemia in newborns [1] that can be differentiated into a diffuse (affecting the entire pancreas), focal, or atypical form (affecting one or multiple smaller regions in the pancreas) [2, 3].

The most frequent genetic causes are mutations in *ABCC8* and *KCNJ11*, coding for the subunits of the ATP-sensitive potassium channel in the beta cell with different modes of inheritance [1–3].

Beckwith-Wiedemann-Syndrom (BWS) is the most common syndrome associated with congenital hyperinsulinism. Other clinical features of BWS include segmental overgrowth, organomegaly, macroglossia, abdominal wall defects and ear and renal abnormalities [2, 4]. Hyperinsulinism in BWS, results from the abnormal regulations of genes on chromosome 11p15, including *ABCC8* and *KCNJ11* [2, 4–7].

Severe and repetitive hyperinsulinaemic hypoglycaemia results in neurological damage and severe developmental retardation [8–10]. Therefore, the main treatment goal is to maintain normoglycaemia. In the majority of CHI patients, this cannot be achieved by dietary measures alone, and drug treatment or pancreatic surgery is necessary [11].

CHI treatment often presents as a dilemma because there is a lack of approved drugs that reduce pathologic insulin secretion for most patients. Diazoxide is the only approved medication for treating CHI in childhood. However, as diazoxide is an agonist of the K_ATP_-channel, patients with homozygous/compound-heterozygous mutations in its coding genes, *ABCC8* and *KCNJ11*, often do not respond to diazoxide. Whereas focal hyperinsulinism can be cured by localizing the affected regions with 18-F-DOPA-PET followed by surgical enucleation, diffuse disease has to be treated with drugs and dietary measures [12–14]. As the only alternative, near-total pancreatectomy can be performed, but it may be ineffective due to persisting hyperinsulinism after surgery. Additionally, it has a high risk of persistent hypoglycaemia or the development of insulin-dependent diabetes mellitus after surgery [15–18]. Therefore, there is an urgent need for new pharmacological treatment options in diffuse CHI. Over the last decades, there have been many case reports describing successful off-label treatment of CHI with somatostatin analogues, such as octreotide and lanreotide [12, 15, 16, 19]. Octreotide can be administered in 4–6 single injections with fluctuating actions [15] or continuously via s.c.-pump. Even with this treatment, hypoglycaemia cannot be prevented for some patients. Pump therapy carries the risk of dislocation of the catheter with subsequent risk for severe hypoglycaemia. Therefore, patients with such severe disease are hospitalized over months.

Longer acting analogues of somatostatin, such as long-acting lanreotide autogel® (LAN-ATG), which has to be administered only every 4 weeks, aim to simplify the treatment and improve the family’s quality of life. These formulations use microspheres of a slowly dissolving polymer or in a supersaturated gel-formulation, providing a predictable pharmacokinetic profile of drug release and steady-state kinetics [20, 21]. Although octreotide and LAN-ATG have a similar activity and affinity profile *in vitro*, there are significant differences with regard to the pharmacodynamics and –kinetics *in vivo* [22]. However, LAN-ATG is approved for the treatment of acromegaly and neuroendocrine tumours (NETs) in adults only [22–24]. Although the mechanism of action is not fully understood, somatostatin analogues promote inhibitory effects on the insulin secretion of beta cells, by binding to specific somatostatin receptors, such as 2 and 5 [24], which address different mechanisms, such as the activation of K^+^/Ca^2+^-channels, inhibitory effects on exocytosis and membrane repolarization [25].

Recently, there have been some reports on the off-label use of LAN-ATG in children with severe CHI [15, 19, 26, 27], leading to increased use in CHI patients, at least in some specialized centres. However, all treated children were at least 7 months or older [15, 19]. To the best of our knowledge, there is no report about LAN-ATG treatment within the first 6 months of life.

Concerns to treat young infants with LAN-ATG might arise from possible adverse effects of somatostatin analogues, particularly gastrointestinal side effects, which are caused by inhibition of gastrointestinal motility, gallbladder contractility and splanchnic blood flow [28]. These side effects include in particular the risk for necrotizing enterocolitis (NEC), and furthermore for gallbladder pathologies and elevated liver enzymes as well as pituitary hormone suppression, which might affect linear growth [22, 27, 29, 30]. However, severe complications of somatostatin analogue treatment, such as NEC, have only been reported in particular cases of neonates with additional risk-factors for NEC, such as low birth weight, cardiac anomalies or prematurity, directly after starting treatment [10, 28]. They were also reported in one case after 2 months of treatment in a child with other risk factors, such as operative bowel manipulation, opiate intake and medical alteration of the intestinal flora [31].

Therefore, we discussed LAN-ATG, as an individual off-label treatment option, with the parents of infants, who had different clinical courses, at 2–3 months of age. The aim of this treatment option was to enable discharge from the hospital without requiring surgical intervention.

## Methods

We report the off-label use of LAN-ATG in 4 infants with congenital hyperinsulinism at different time intervals between 2011 and 2016, who did not sufficiently respond to their current nutritional and drug treatment (Table 1). CHI was due to homozygous *ABCC8* gene mutations (*n* = 2) or Beckwith-Wiedemann-syndrome (BWS) (*n* = 2). All participants received an initial treatment with diazoxide (dosage interval: 10–14 mg/kg /day) and a diet enriched with 20% maltodextrin. Because diazoxide did not provide sufficient glycaemic control, patients #1, #2 and #4 were treated with subcutaneous (s.c.) octreotide (dose range: 36 to 43 μg/kg/d), continuous s.c. glucagon infusions (dose range: 50 to 84 μg/kg/d), and/or i.v. glucose infusion. In this way, symptomatic hypoglycaemia was prevented and glycaemic control could be improved. However, all four patients had at least one (asymptomatic) blood glucose level < 1.7 mmol/l per week and at least three blood glucose levels between 1.7 and 2.8 mmo/l per week, making discharge from inpatient treatment impossible.

**Table 1:** Summary of all four patients with medical conditions, treatment plan and observed side effects

Clinical characteristics	Patient #1	Patient #2	Patient #3	Patient #4
Age at presentation	hypoglycaemia DOL 1	hypoglycaemia DOL 1	hypoglycaemia at birth	hypoglycaemia DOL1
Mutation	homozygous c.563A > G (ABCC8)	homozygous c.1176G > C (ABCC8)	mosaic paternal UPD 11	mosaic paternal UPD 11
Associated syndromic condition	no	no	yes, BWS	yes, BWS
Treatment prior to LAN-ATG	DZ p.o. 10 mg/kg/d OCT sc. 43 μg/kg/d GLUC sc. max. 50 μg/kg/d carbohydrates 154 g/d p.o.	DZ p.o. 14 mg/kg/d OCT sc. 36 μg/kg/d GLUC sc. max. 84 μg/kg/d carbohydrates 100 g/d p.o.	DZ p.o. 10 mg/kg/d carbohydrates 69 g/d p.o.	DZ p.o. 10 mg/kg/d OCT sc. 40 μg/kg/d carbohydrates 78 g/d p.o. and i.v.
LAN-ATG treatment	30–90 mg/month (4.8–6.9 mg/kg)	30–90 mg/month (5–7.5 mg/kg)	1× 30 mg (5.3 mg/kg)	60 mg/14d-90 mg/month (9.5–13.6 mg/kg)
Carbohydrates after LAN-ATG	64.2 g/d p.o.	63.6 g/d p.o.	63 g/d p.o.	78 g/d p.o.
Duration of LAN-ATG treatment	25 months	23 months	single injection	3 months
Additional medication	none	none	none	DZ 8 mg/kg/d
Side effects of LAN-ATG	nodules at injection site pre-existing cholelithiasis	nodules at injection site pre-existing biliary sludge	none	nodules at injection site

*DOL* days of life, *UPD* uniparental disomy, *BWS* Beckwith Wiedemann syndrome, *DZ* Diazoxide, *OCT* octreotide, *GLUC* glucagon, and *LAN-ATG* lanreotide autogel

Aware of the severe hyperinsulinism that accompanies a high risk of severe hypoglycaemia, we decided, after careful discussion with the parents of possible risks and benefits, to perform LAN-ATG treatment. The clinical situation was different in each of the four patients but the main aim was to try LAN-ATG off-label as an alternative to surgery. The ethical review committee at our institution was informed of the expanded access with LAN-ATG as an individual attempt. After parents gave their written informed consent, each patient was injected with LAN-ATG via deep subcutaneous injection after the local application of analgesic lidocain/prilocain-patches. Table 1 summarizes the clinical course, treatment, and observed side effects for all four patients. None had significant risk factors, such as a low birth weight or prematurity, for developing NEC.

The mean blood glucose levels (mean of 10.22 measurements per day, range of 4–22), frequency of hypoglycaemia and carbohydrate intake were evaluated within 7 days prior and 7 days after the first LAN ATG injection.

Continuously maintaining the blood glucose over the international hypoglycaemia threshold of 3.9 mmol/l [32] is often impossible in patients with severe hypoglycaemia when using conservative treatment measures. However, adapting the target range to >3.3 mmol/l and accepting infrequent occurrence of short episodes of hypoglycaemia between 2.8 and 3.3 mmol/l often still provides a sufficient safety margin for preventing severe hypoglycaemia and its consequences. Therefore, we used these limits as treatment targets in these patients and primarily aimed for consequent prevention of blood glucose < 2.8 mmol/l as well as severe/symptomatic hypoglycaemia prior to discharge.

Side effects were monitored using ultrasound, length-growth measurements and laboratory tests over a total of 52 treatment months. Because of the very small number of patients and their clinical and therapeutic heterogeneity, statistical analyses and comparisons could not be performed, in retrospect, in all of the evaluated parameters. The aim in each patient was to identify the best treatment mode in terms of the diet (normal carbohydrate intake and fasting tolerance), medication (dosage) and glucose level with an acceptance of occasional episodes of mild asymptomatic hypoglycaemia (Table 1).

## Results

### Mean blood glucose concentration and frequency of hypoglycaemia before and after the first LAN-ATG application

**Fig. 1:**
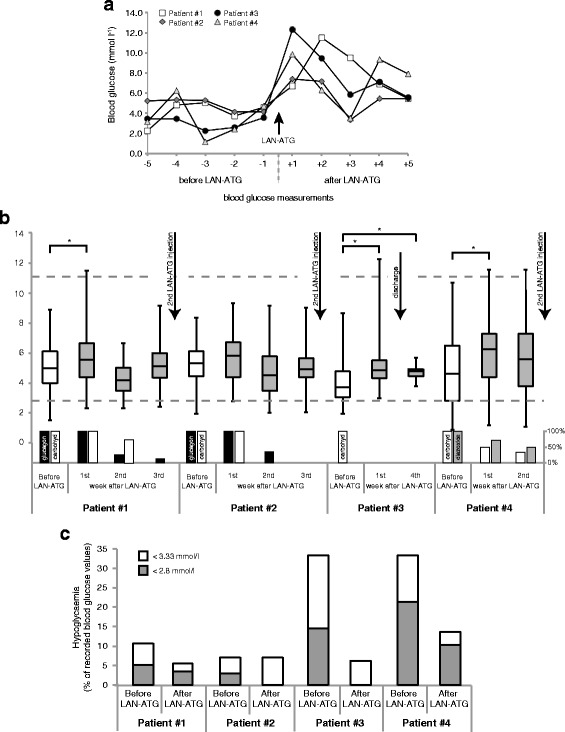
**a** Blood glucose levels on the first day of LAN-ATG administration. **b** Mean blood glucose within 7 days before and 2 to 4 weeks after the first injection of LAN-ATG. During this phase there was an individual adjustment of therapy with discontinuation or reduction of glucagon and/or diazoxide and additional carbohydrate supply. Glucagon/diazoxide and additional carbohydrate treatment, shown as 100% column at LAN-ATG injection, with weekly mean after LAN-ATG (column 0–100%). The second LAN-ATG injection was done between 2 and 4 weeks, based on individual clinical decisions. * Significance determined by Student’s t-test. *p < 0.05. **c** Percentage of blood glucose < 3.3 mmol/l among all recorded blood glucose values within 7 days before and 7 days after the first injection of LAN-ATG

Shortly after the first injection of LAN-ATG, all patients had a pronounced increase of blood glucose levels at least > 7.2 mmol/l (within 1–2 h) (Fig. 1a) which returned to normal within the first day. In all patients, the mean blood glucose level was higher over the period of 7 days after the injection of LAN-ATG compared to the period of 7 days before treatment (Fig. 1b). The mean increase of randomly measured blood glucose concentrations for all patients was 0.75 mmol/l (0.39–1.19 mmol/l). The frequency of blood glucose < 2.8 mmol/l could be slightly lowered by a mean of 7.7% (1.7–14.81%), and the rate of blood glucose concentrations < 3.3 mmol/l could be decreased by an average of 13% (0.1–27.1%). Both effects were more pronounced in both patients with BWS (#3 and #4) compared to those with *ABBC8* mutations (Fig.1c). Over the following weeks in particular intravenous treatment (glucose, glucagon) and dietary treatment was subsequently reduced to allow discharge with the minimum of additional carbohydrates compared to dietary recommendations without a substantial worsening of hypoglycaemia rate or severity. Therefore, LAN-ATG was regarded beneficial in all patients to improve glucose homeostasis.

### Carbohydrate intake

**Fig. 2:**
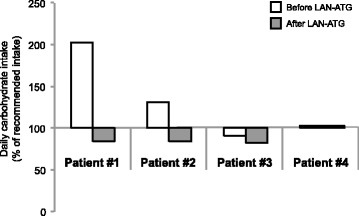
Mean carbohydrate intake within 7 days before and 7 days after the first injection of LAN-ATG in % of the daily recommendation according to the German nutrition society(27)

After LAN-ATG injection, the carbohydrate requirement to prevent severe hypoglycaemia decreased in patient #1–3 (Fig. 2). The mean reduction was 7 g/kg body weight/d (1.7–12.8 g/kg body weight/d). In patient #4, there was no absolute reduction in the quantity of daily carbohydrates; however, there was a shift from intravenous glucose to oral carbohydrate uptake. Patients #1 and #2 could especially be significantly reduced to an age-appropriate carbohydrate intake, as recommended by the German nutrition society [33], which may explain the less marked reduction in the occurrence of hypoglycaemic episodes and the lower mean of blood glucose concentrations in the following weeks in these patients compared to children with BWS.

### Adverse events

**Fig. 3:**
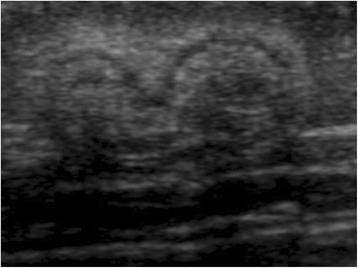
Subcutaneous induration nodules

Patient #1 was diagnosed with asymptomatic gallstones and patient #2 presented with biliary sludge by abdominal ultrasound before the first LAN-ATG injection, which was presumably due to prior octreotide treatment. They were treated with ursodesoxylcholic acid. In patient #2, the sludge resolved within 2 months, whereas gallstones persisted in patient #1 without further progression in size and number or any clinical symptoms. There were no clinical or laboratory signs of cholestasis. All patients showed a normal length growth within their respective target ranges. No acute gastrointestinal side effects occurred. All patients receiving repetitive LAN-ATG injections developed subcutaneous nodules at the injection sites, which showed no signs of inflammation or tenderness (Fig. 3). No further side effects were observed. Both patients with BWS developed typical tumours, such as liver haemangioma and neuroblastoma, which are linked more to the primary disease than to LAN-ATG treatment.

### LAN-ATG injection and dosage adaption over time

Patient 3 just needed a single injection until remission (5.3 mg/kg). Patients 1 and 2 were treated every 3–4 weeks for a mean period of 24 months, whereas patient 4 received the injection every 2 weeks for 3 months (dose range: 4.8 to 19 mg/kg/month). The age at the first LAN-ATG injection was 7.5 to 9 weeks. An increased frequency of hypoglycaemia was observed shortly before the next injection in all except patient #3, who only needed a single injection to remain normoglycaemic. The remaining three patients required additional injections in the following month because of recurrent blood glucose measurements that were below the target range, mostly at a level between 2.2 and 2.8 mmol/l, five to nine times per week. The dose for patient #4 was increased only once; those for patients #1 and #2 had to be increased twice. Dose increases were performed using 30-mg increments. In patients #1 and #2, all concomitant drugs, such as glucose infusions, octreotide, diazoxide, or continuous glucagon administration, could be successfully stopped after the first LAN-ATG injection. Patient #3 was not receiving any other medications at the time of the first LAN-ATG injection. After LAN-ATG application, all four patients could be discharged from hospital care, 2–3 month after the first injection. This amount of time was needed to find the right dosage, since we started with a low dosage for safety reasons.

## Discussion

We evaluated the off-label use of LAN-ATG in four infants <6 months of age with severe congenital hyperinsulinism. The aim of the treatment was to stabilize glucose homoeostasis and fasting tolerance, to achieve an appropriate carbohydrate intake and to reduce the parenteral co-medication, which would allow for discharge from the hospital without requiring pancreatic surgery. LAN-ATG has not been used so far in early infancy, since it was uncertain, which effects could be expected. Once the drug has been injected, the pharmaceutical effect will last for at least 4–6 weeks. Potentially this might lead even to hyperglycaemia and the need of following insulin therapy to overcome the inhibitory effect on insulin secretion. However, we were most concerned with the risk to develop necrotizing enterocolitis, without the chance to discontinue the lanreotide action.

On the other hand NEC was almost always reported in patients with additional risk factors. The good experiences with octreotide encouraged us to use lanreotide, based on the limitations of octreotide with frequent injections or continuous subcutaneous administration with an insulin-pump. Furthermore those parents with older CHI patients, who were switched from octreotide to LAN-ATG experienced this change as a major improvement in the quality of life, as they told us in personal communication.

In one patient with BWS, there was no further hypoglycaemia after a single injection. We assume that this has to be attributed mainly to spontaneous remission of the disease, after a period of blood glucose stabilization, which was perhaps facilitated or bridged by LAN-ATG treatment. In three patients, significant improvement in glycaemic control was achieved within the first week after LAN-ATG injection with less frequent and less pronounced hypoclycaemia than before. Subsequently, the concomitant drug and dietary treatment could be reduced in all patients, without substantial worsening of glycaemic control. In both patients with CHI due to K_ATP_-channel mutations with increased carbohydrate requirements before LAN-ATG treatment, intake could be reduced to normal after the first injection and all other concomitant drugs could be stopped, which explains the less marked reduction in the frequency of hypoglycaemic episodes and the lower mean of the blood glucose level in the second and third week after the injection. Patient #3 continued to take diazoxide, as he was partially responsive to it. Most importantly, all parenteral medication could be stopped, allowing the discharge of all patients to home-care for the first time.

However, in all patients, we were still unable to maintain blood glucose continuously >3.9 mmol/dl. Using LAN-ATG, only one injection per month was needed instead of 3–6 injections daily or continuous s.c. application of octreotide. The latter one has the risk of catheter dislocation, resulting in severe hypoglycaemia. With the use of local anaesthetics, the injection of LAN-ATG was reasonably tolerated without prolonged crying or anxiety of the infants. We observed only mild side effects, such as palpable subcutaneous nodules at the injection site, and mild gastrointestinal discomfort, such as flatulence and diarrhoea; the latter disappeared after the first weeks of LAN-ATG use.

Of note, we discussed LAN-ATG with the parents of infants who had severe forms of congenital hyperinsulinism and usually do not respond sufficiently to all other pharmacological treatment options (e.g., diazoxide and octreotide) as well as suffered from recurrent severe hypoglycaemic events and were therefore hospitalised over months. In each of the four patients, LAN-ATG use was an individual decision, made with the parents after careful discussion, as the last attempt for conservative treatment before surgery. The aim of treatment was to allow for discharge from the hospital. The individual treatment goal for these patients was not the complete prevention of any hypoglycaemic event; instead, it was sufficient stabilization, making home-care management tolerable with a tolerable risk profile for severe hypoglycaemia in this setting. Therefore, we considered the off-label use in all four patients on an individual basis as successful addressing this aim, although episodes of hypoglycaemia could not completely be prevented even after LAN-ATG injection. Despite the descriptive nature of our data, these may provide an exploratory basis for future studies following more standardized protocols for treatment and evaluation of efficacy and safety. For such future studies we would recommend the use of continuous glucose monitoring.

The improved effect of LAN-ATG over octreotide might be a dose-dependent effect. We used a higher monthly dose of LAN-ATG compared to the prior cumulative monthly octreotide dosage. However, although both substances have a similar activity and affinity profile, there are slight differences in the pharmacodynamics and –kinetics, limiting dose comparability between both formulations.

LAN-ATG treatment efficacy is likely to vary with the underlying genetic defect/aetiology of CHI. Here, the two patients with BWS had a more pronounced reduction of hypoglycaemia and higher mean blood glucose concentration. Both infants with homozygous *ABCC8* mutation had a higher reduction in the daily carbohydrate intake. However, these differences might be coincidental because of the small number of cases.

Our findings are consistent with reports of successful LAN-ATG use in much older children with CHI [15, 19, 26]. However, larger and more standardized prospective studies are needed to evaluate the efficacy and safety of LAN-ATG compared with other treatment options with the overall aim of achieving approval. Today, LAN-ATG treatment for CHI might be an individual approach in some children with inadequate response to approved therapy. In our opinion, the use of LAN-ATG should not be considered due to the long half-life and potential risk of NEC in the neonatal period. If urgently needed, it will be reasonable to start with a low-dose of off label octreotide after the first weeks of life, which has a short half-life and can be discontinued immediately in the event of severe side effects, such as NEC. It has been used in many infants with CHI with a good safety profile [34, 35]. If increasing doses of octreotide are sufficiently tolerated and effective, LAN-ATG may be considered after the first months of life when important treatment goals, such as discharge from the hospital cannot be achieved, and otherwise near total pancreatectomy would be necessary. Up to now, there are insufficient long-term safety data regarding, for example, pituitary dysfunction and the development of insulin resistance. Off -label use of sirolimus might be an alternative to LAN-ATG in infancy, with a completely different and substantial risk profile and variable results in children with CHI and persistent hypoglycaemia [36, 37].

## Conclusion

Based on these experiences, LAN-ATG might be a treatment option already in early infancy to allow discharge from hospital or omit pancreatic surgery. However, because safety data remain scarce, the application should be strictly limited to otherwise refractory cases without NEC risk factors, and requires careful counselling of the parents about chances and risks of this treatment option. If successful, only one injection per month of LAN-ATG provides higher flexibility in everyday life than octreotide.
